# The Value of MRI in the Diagnosis of Primary Biliary Cirrhosis and Assessment of Liver Fibrosis

**DOI:** 10.1371/journal.pone.0120110

**Published:** 2015-03-17

**Authors:** Ying Meng, Yuting Liang, Mingming Liu

**Affiliations:** 1 Department of Radiology, Beijing Obstetrics and Gynecology Hospital, Capital Medical University, Beijing, China; 2 Department of Radiology, Beijing Friendship Hospital, Capital Medical University, Beijing, China; Emory University School of Medicine, UNITED STATES

## Abstract

**Objectives:**

To evaluate MRI findings in patients with primary biliary cirrhosis (PBC) and to determine the value of MRI in the diagnosis of PBC and assessment of liver fibrosis.

**Materials and Methods:**

This study reviewed the prevalence of MRI abnormalities seen in 45 PBC patients in the past four years, including 33 patients who underwent liver biopsy. Correlation between the MRI findings and the pathological stage was determined.

**Results:**

There were 33 patients who underwent liver biopsy. Twenty-five patients (75.8%) had non-homogeneous changes in the liver signal intensity, 25 (75.8%) had a periportal halo sign, and 29 (87.9%) had lymphadenopathy. The short axis of the enlarged lymph nodes was a mean of 1.2±0.3 cm. A strong positive correlation was observed between histological stage and the inhomogeneity of liver signal intensity (P<0.001). There were significant differences among the four histological stages based on the periportal halo sign (P=0.034), and the grading of the periportal halo sign was found to be significantly correlated with the histological stage (P<0.001). Grading of the periportal halo sign was significantly different at stage II versus III, and stage III versus IV; no significant difference was found between stages I and II. There were also no significant differences among the four histological states in the occurrence and size of enlarged lymph nodes (P=0.674 and P=0.394).

**Conclusion:**

MRI is valuable in the diagnosis of PBC, and the periportal halo sign and liver signal intensity help to evaluate the degree of liver fibrosis.

## Introduction

PBC is a chronic cholestatic liver disease, resulting from genetic and environmental factors [1], and it mainly affects mature women[2,3]. The prevalence of PBC varies internationally and regionally[4]; the annual incidence rates vary from 0.7 and 49 cases per million people and prevalence rates range between 6.7 and 402 cases per million people[5]. Pathologically, PBC is characterized by chronic nonsuppurative destructive cholangitis (CNSDC) and destruction of interlobular bile ducts. It results in progressive accumulation of fibrous tissue and eventually development of micro- or macro-nodular cirrhosis. The most common symptoms of PBC are fatigue and pruritus [6,7]. Ursodeoxycholic acid (UDCA) is currently the only FDA-approved medical treatment for PBC, and liver transplantation is performed in patients with advanced-stage PBC [8]. Serum anti-mitochondrial antibodies (especially AMA-M_2_) are pathognomonic in PBC, and are present in more than 95% of patients [9]. Liver biopsy is the gold standard test [10], and it is required in patients with AMA negativity. However, liver biopsy is an invasive procedure, so repeating this test over time in an individual patient can be difficult and not well received. In addition, sampling errors can occur because liver biopsies only sample a small part of the liver. Finally, histological examination is prone to intraobserver and interobserver variation[11]. MRI is a noninvasive diagnostic tool, and early detection and diagnosis of PBC using MRI may play an important role in developing a treatment plan, monitoring the severity and progression of the disease, and assessing patient prognosis.

Compared with other diffuse liver diseases, PBC has common MRI findings (such as a change in the liver signals, morphology and portal hypertension), and unique signs. Wenzel et al. (2001) [12] first suggested that the periportal halo sign was a specific sign of PBC. Kovač et al. (2012) [13] pointed out that abdominal lymphadenopathy was another characteristic MRI finding in PBC patients. This study was conducted to further determine the value of MRI in PBC diagnosis, and especially to highlight the value of its assessment of liver fibrosis and to provide a basis for the use of MRI as a noninvasive method to monitor the curative effect and prognosis of PBC.

## Materials and Methods

### Patients

A diagnosis of PBC is made when two of the following three criteria are met[14]: (1) biochemical evidence of cholestasis, based mainly on alkaline phosphatase elevation; (2) presence of anti-mitochondrial antibody (AMA); and (3) histological evidence of nonsuppurative destructive cholangitis and destruction of interlobular bile ducts. Cases were excluded when patients were diagnosed with viral hepatitis, alcoholic hepatitis, fatty liver, autoimmune hepatitis (AIH), primary sclerosing cholangitis (PSC) or other kinds of diffuse liver diseases, and patients with coexisting PBC or any of above diseases.

The study was approved by the ethics committee of Beijing Friendship Hospital and the ethics committee of Beijing Obstetrics and Gynecology Hospital, Capital Medical University. Written informed consent was obtained from all participants and all data has been anonymized and de-identified. Forty-five patients in the two above mentioned hospitals from 2009 to 2013 who met the above standards and who had undergone MRI during this time were included in this retrospective study. There were 38 women and 7 men ranging from 33 to 77 years old (mean age 51.3±9.7 years). There were 33 patients who underwent liver biopsy in the group, including 27 women and 6 men ranging from 35 to 68 years old (mean age 50.7±8.8 years). The time between liver biopsy and MRI scan ranged from 15 to 123 days (mean time 61 days). Biopsy results were analyzed by an experienced histopathologist who was blinded to the clinical results. Histological stage was determined according to the Ludwig’s classification [15]: stage I (cholangitis stage); stage II (bile duct hyperplasia stage); stage III (fibrous stage); and stage IV (cirrhotic stage). The histological stages of the 33 PBC patients are presented in Table 1.

**Table 1:** Histological stages in PBC patients.

Histological stage	n	Percentage (%)
I	6	18.2
II	12	36.3
III	9	27.3
IV	6	18.2
Total	33	100

### MR examination

Patients fasted from solids and liquids 4–6 hours before the MRI scan. MRI examinations were performed using a 3.0 Tesla magnet (Signa Excite HD, General Electric Medical Systems, USA) with an 8-element phased-array abdominal coil. Routine breath holding in-phase and out-of-phase axial T1-weighted images and T2-weighted images were acquired. T1-weighted imaging was performed using a fast spoiled gradient echo sequence. The imaging parameters were as follows: TR, 224 ms; TE, 2.7 ms; number of slices, 20; slice thickness, 7 mm; matrix size, 271×192 pixels; field of view, 38 cm×38 cm; and average acquisition time, 18s. T2-weighted imaging was performed using a fast spin echo sequence. The imaging parameters were as follows: TR, 6000 ms; TE, 107/Ef ms; number of slices, 20; slice thickness, 7 mm; matrix size, 288×224 pixels; field of view, 38 cm×29 cm; and average acquisition time, 2 min.

### Image analysis

A retrospective evaluation of PBC patients was performed independently by two radiologists with over 5 and 10 years’ experience in abdominal MRI diagnosis. The abnormal MRI signs found in the study were summarized as general signs and specific signs of PBC. General signs refer to common signs of liver fibrosis and liver cirrhosis that have various causes, and specific signs refer to signs that are not seen or rarely seen in other common liver fibrosis (for example, viral fibrosis, alcohol liver fibrosis). The standards for abnormal signs include: 1) diffuse hepatomegaly[13] (defined as craniocaudal diameter in the medioclavicular line ≥15.5 cm); 2) splenomegaly (edge of the spleen over the edge of the liver or spleen rib lateral margin of five or more units); 3) portal hypertension, defined as an increased portal vein diameter (the diameter>1.3 cm), portosystemic collaterals, and ascites; 4) periportal hyperintensity[13] (T2-weighted hyperintensity around portal venous branches); 5) periportal halo sign[12] (T1- and T2-weighted low signal intensity lesion, centered around portal venous branches, 5 mm-1 cm in size); and 6) lymphadenopathy[16]: measuring the short axis of lymph nodes, mild (1.0–1.5 cm), moderate (1.6–2.0 cm), or marked (>2 cm). Each MRI sign for each patient was recorded as with or without.

In addition, without pathological stage information, non-homogeneity of liver signal and periportal halo sign were graded according to the extent and degree or quantity. The criteria were as follows: 1) non-homogeneity of liver signal: 0, no obviously abnormal signal; 1, slightly uneven or partially increased signal located in any liver segment; 2, diffuse slightly uneven increased signal or partially obviously uneven signal; and 3, diffused and obviously uneven signal; and 2) periportal halo sign: 0, none; 1, limited and few; 2, scattered with medium quantity; and 3, diffused with many halo signs.

The differences between the two observers were resolved by consensus, and the observations were recorded.

### Statistics

All analyses were performed using SPSS 19.0 software (IBM, New York, US). Continuous variables were described using the mean ± standard deviation (SD) while categorical and ordinal variables were presented as frequencies. Analysis of variance (ANOVA) was used to compare the size of enlarged lymph nodes stratified according to the individual histological stage. The occurrence of a periportal halo sign and enlarged lymph nodes between each pathological group were analyzed using the rank sum test. The grading of the liver signal non-homogeneity and a periportal halo sign were ranked data. Correlation was assessed using the Spearman correlation coefficient. The statistical significance level (P value) was set at 0.05.

## Results

The clinical characteristics of the 45 PBC patients enrolled in our study are presented in Table 2.

**Table 2:** Clinical characteristics of the 45 PBC patients.

Symptoms	n (%)	Laboratory Results	n (%)
Liver function abnormality	29 (64.4)	AMA-M2 positive	36 (80.0)
Pruritus	15 (33.3)	Alkaline phosphatase elevation	40 (88.9)
Fatigue	15 (33.3)	Gamma-glutamyl transpeptidase elevation	41 (91.1)
Jaundice	12 (26.7)	Alanine aminotransferase elevation	36 (80.0)
Sjogren's syndrome	10 (22.2)	Aminotransferase elevation	36 (80.0)
Right epigastric discomfort	9 (20.0)	Total bilirubin elevation	26 (57.8)
Anorexia, loss of appetite	5 (11.1)		

### PBC MRI signs

General signs included diffuse hepatomegaly, splenomegaly, widened portal vein diameter, portosystemic collaterals, ascites, liver signal intensity T2-weighted non-homogeneous change, and periportal T2-weighted hyperintensity. Specific signs included periportal halo sign and lymphadenopathy (Figs. 1–8) (Table 3).

**Fig 1:**
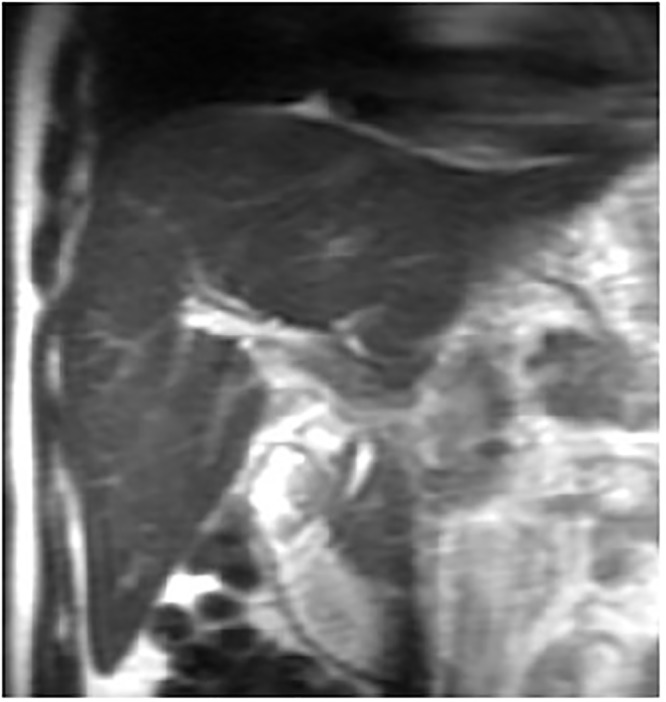
male, 61 years, diffuse hepatomegaly shown on coronal T2-weighted image.

**Fig 2:**
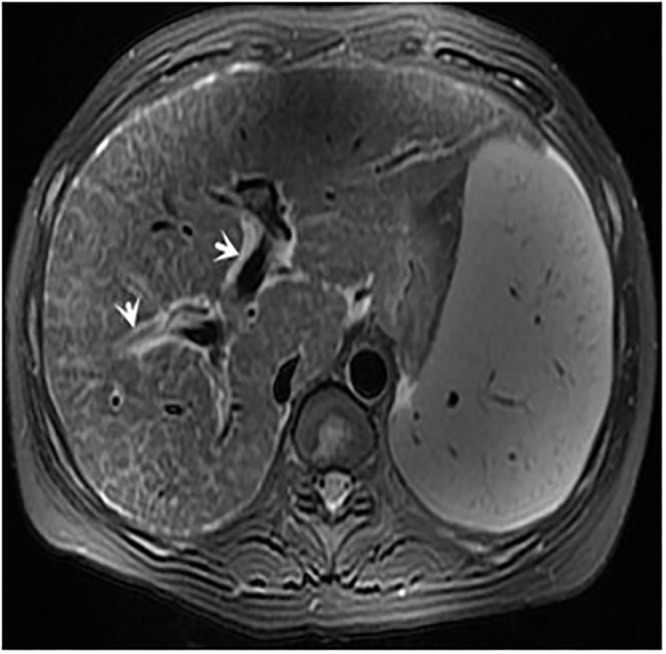
female, 42 years, axial T2-weighted image shows splenomegaly and demonstrates periportal hyperintensity around medium-sized portal triads (*arrows*).

**Fig 3:**
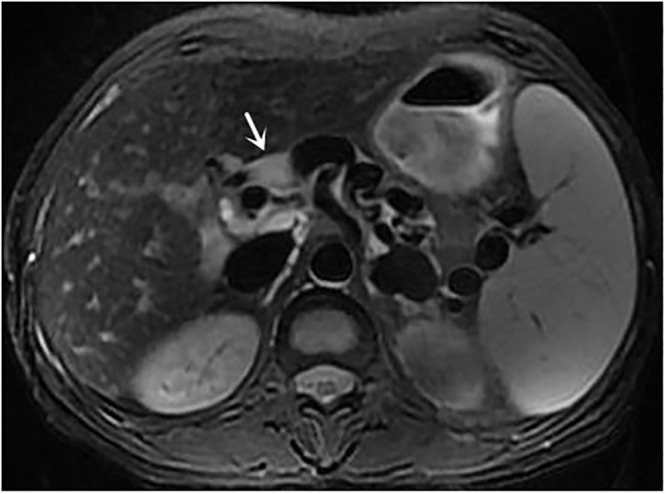
female, 52 years, axial T2-weighted MRI shows lymphadenopathy (*arrow*), and portosystemic collaterals are shown as areas without signal intensity.

**Fig 4:**
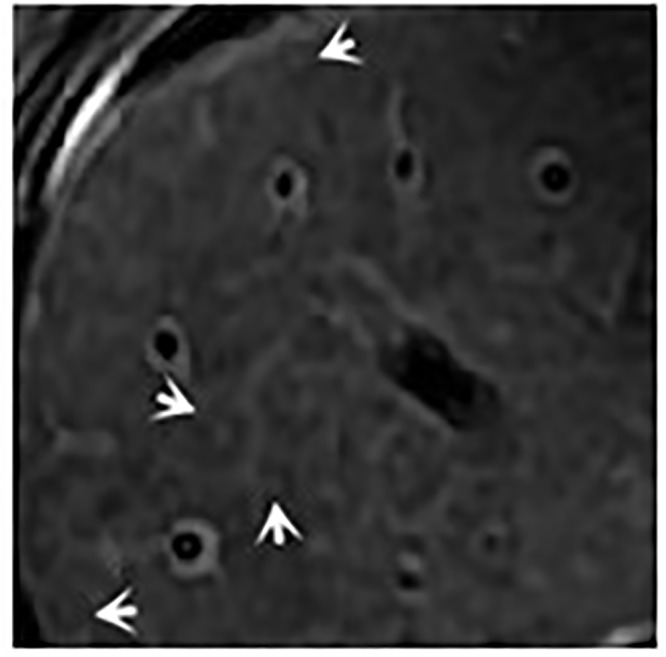
male, 61 years, axial T2-weighted image shows periportal halo signs as hypointense areas around the portal veins (*arrows*).

**Fig 5:**
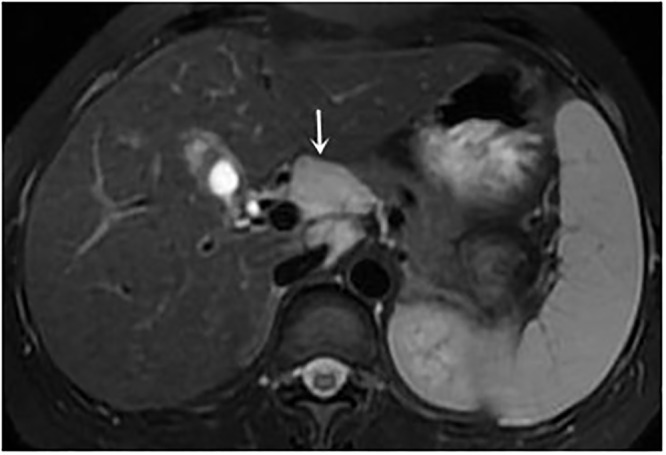
female, 42 years, axial T2-weighted MRI shows lymphadenopathy (*arrow*).

**Fig 6:**
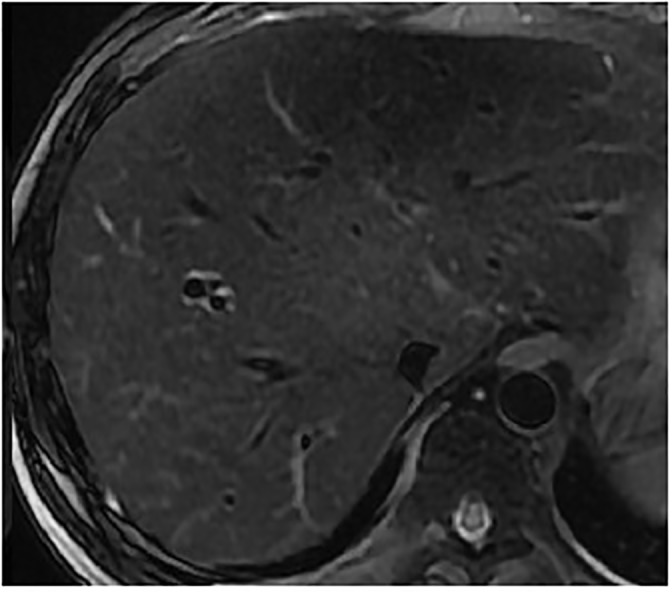
male, 37 years, stage I with signal grade 1 and halo sign grade 1.

**Fig 7:**
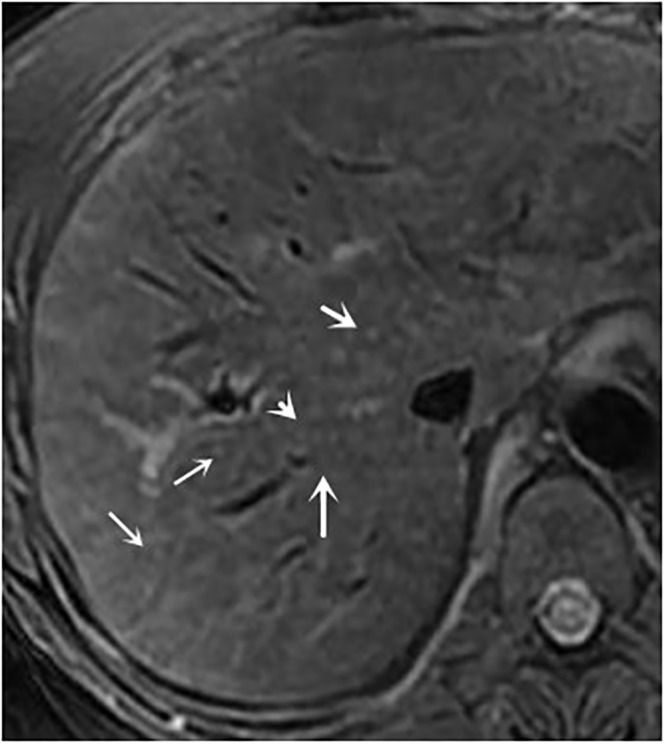
female, 50 years, stage II with signal grade 2 and halo sign grade 2.

**Fig 8:**
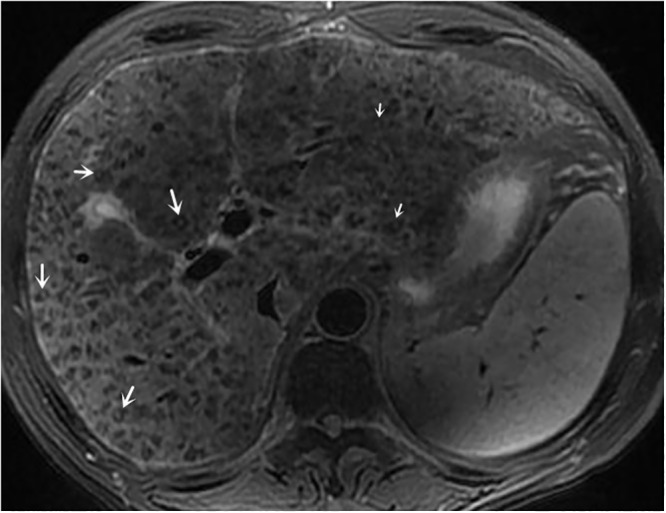
male, 54 years, stage IV with signal grade 3 and halo sign grade 3.

**Table 3:** MRI findings.

MRI signs		n	Percentage (%)
**General signs**			
Diffuse hepatomegaly		17	37.8
Splenomegaly		45	100
Portal hypertension	Increased portal vein diameter	27	60
	Portosystemic collaterals	4	8.9
	Ascites	25	55.6
Liver signal intensity T2-weighted non-homogeneous change		34	75.6
Periportal T2-weighted hyperintensity		35	77.8
**Specific signs**			
Periportal halo sign		34	75.6
Lymphadenopathy		39^※^	86.7

^※^There were 92 enlarged lymph nodes in 39 (86.7%) patients. The short axis was a mean of 1.3±0.4 cm. There were 79 (85.8%) mildly enlarged, 10 (10.9%) moderately enlarged, and 3 (3.3%) markedly enlarged lymph nodes. The specific distribution of enlarged lymph nodes is presented in Table 4.

**Table 4:** Specific distribution of enlarged lymph nodes.

Location	n	Percent (%)
Portacaval	30	32.60
Porta hepatis	18	19.60
Peripancreatic	18	19.60
Retroperitoneal	15	16.30
Aortocaval space	6	6.50
Gastrohepatic ligament	5	5.40
Total	92	100

### Correlation between the MRI findings and the degree of liver fibrosis

Periportal hyperintensity on T2-weighted images was observed in 25 of the 33 patients (75.8%), with 100% at stage I, 75% at stage II, 88.9% at stage III, and 33.3% at stage IV.

**Fig 9:**
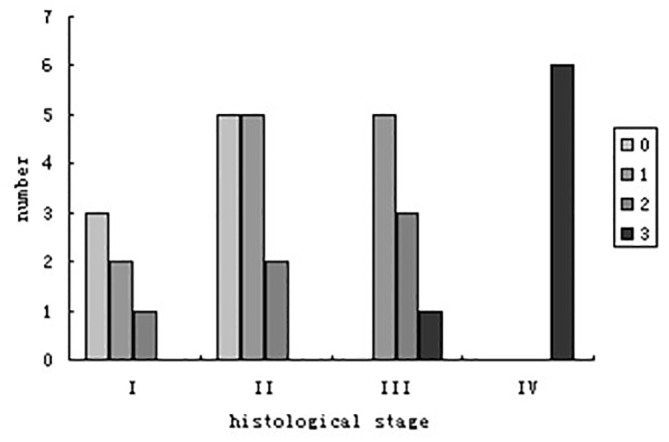
Liver signal grade at different histological stages.

Degree of liver signal intensity was assessed according to the standard. Homogeneous liver signal intensity was observed in 8 patients (24.2%) at stage I and stage II, and 25 patients (75.8%) had non-homogeneous liver signal changes. Patients at stage I, II and III mainly showed signal grade 1 and 2, but patients at stage IV all had signal grade 3. A strong positive correlation was observed between histological stage and the non-homogeneity of liver signal intensity (r = 0.703, P<0.001)(Fig. 9).

**Fig 10:**
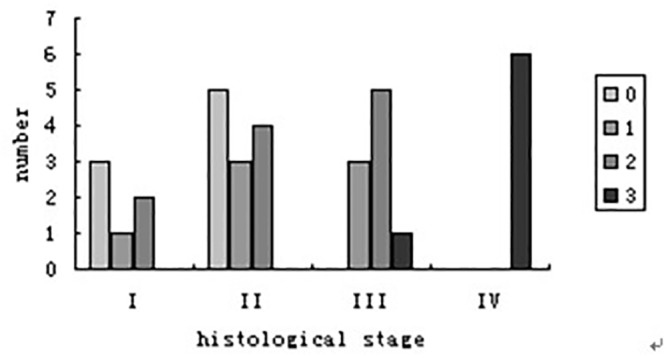
Halo sign grade at different histological stages.

The degree of the periportal halo signs was assessed and observed in 25 patients (75.8%). Eight patients (24.2%) were grade 0, with 3 at stage I and 5 at stage II. Patients at stage I and II mainly showed halo sign grade 0 and 1, patients at stage III mainly showed grade 2, and patients at stage IV all showed grade 3. There were significant differences among the four histological stages based on the periportal halo sign (P = 0.034), and the periportal halo sign grading was found to be significantly correlated with histological stages (r = 0.687, P<0.001)(Fig. 10). The periportal halo sign grading was significantly different at stage II versus III, and stage III versus IV. No significant difference was found between stages I and II.

Seventy enlarged lymph nodes were observed in 29 patients (87.9%). The short axis of enlarged lymph nodes was a mean of 1.2±0.3 cm. There was no significant difference among the four histological stages (P = 0.674 and P = 0.394) for either the occurrence or size of the enlarged lymph nodes. The situation of lymphadenopathy at different histological stages is presented in Table 5.

**Table 5:** The situation of lymphadenopathy at different histological stages.

Histological stage (n)	Patients (numbers of enlarged lymph node)	Percentage (%)	Short axis mean (cm)
I (6)	5 (12)	83.3	1.2±0.2
II (12)	10(20)	83.3	1.3±0.2
III (9)	9 (28)	100.0	1.3±0.3
IV (6)	5 (10)	83.3	1.1±0.2
Total (33)	29 (70)		

Note: percentage = cases of patients / total number of patients with the corresponding histopathology.

## Discussion

### Pathological basis of PBC MRI general signs

Diffuse hepatomegaly was the main pattern of liver morphological changes in PBC[17], and this was related to hyperplasia of the bile duct and cholestasis. This is different from viral hepatitis, where liver volume atrophy is more common. In this study, diffuse hepatomegaly was observed in 37.8% of PBC patients in all histological stages, similar to the 40.9% prevalence reported by Kovač et al[13], but patients with mild hepatomegaly, for example, an indistinct liver margin, would be excluded based on our standards. Further research should include measuring the liver volume.

Portal hypertension is a common sign of PBC patients, including splenomegaly, varices and ascites. In this study, splenomegaly was observed in all patients, portosystemic collaterals in 8.9% of patients and a small amount of ascites in 55.6% of patients. However, none of the patients had hepatocellular carcinoma, cholangiocarcinoma or any other malignant diseases.

The sign of periportal hyperintensity is associated with periportal edema, inflammatory cell infiltration and dilatation of lymph vessels[18], which can be seen in many kinds of diffuse liver diseases. However, PBC patients have higher rates of this sign in the MR images. The incidence was reported as 100% at stages I and II, 75% at stage III, and 33% at stage IV by Kobayashi et al[19]. Our study has favorable results with 100% at stage I, 75% at stage II, 88.9% at stage III, and 33.3% at stage IV. The frequency of periportal hyperintensity is highest at stage I, because this sign parallels the incidence of inflammation in each stage. It was confirmed that inflammation in periportal spaces persists with disease progression, although liver fibrosis is the prime manifestation in advanced stages. This explains why periportal hyperintensity still has a high rate in later stages, but lower than in the earlier stages[20].

### Clinical relevance of MRI specific sign with PBC pathological changes

The periportal halo sign showed numerous lesions involving all hepatic segments without occupy affecting. The periportal halo sign is caused by fibrous tissue deposition and hepatocellular parenchymal extinction around the portal triads. The periportal halo sign was more obvious in T2-weighted images. The periportal halo sign resembles a "target" sign with a ring-form hypointensity around a punctate hyperintensity which represents the small branches of the portal vein presenting as punctate hyperintensity without flow-empty effects. It is necessary to distinguish this finding from regenerating nodules, which are not uniform size, may show occupying effect, and have various signal intensities[21]. but are conspicuously low in T2-weighted images with the accumulation of iron[22], and are not located around portal venous branches. The periportal halo sign was seen in 75.8% of patients in the 33 patients, and its prevalence in the four stages (stage I, II, III, and IV) was 50%, 58.3%, 100%, 100%, respectively. There were significant differences among the four histological stages based on the periportal halo sign (P = 0.034), and these results are similar to the finding of Kovač et al[13]. who reported periportal halo sign in 14.3% of PBC patients at stage I, 33.3% at stage II, 100% at stage III, and 85.7% at stage IV.

The periportal halo sign is thought to be a unique sign for PBC. However, our study first defined the grade of the periportal halo sign, and non-homogeneity of liver signal was also graded. The correlation between the two types of grading and the degree of liver fibrosis was calculated. The degree of liver fibrosis is often used to indicate the severity of PBC, which is important to monitor the curative effect and prognosis of PBC. There were a large number of halo signs involving all the hepatic segments, and non-homogeneity of liver signal was more diffuse and evident at later stages; a strong positive correlation was observed between the histological stage and the two signs. Cholangitis and bile duct hyperplasia are the major pathological changes in the early stage of PBC, which does not cause many halo signs in MR images. As the disease progresses, the dominant histology is progressing fibrosis and focal hepatocyte necrosis, which leads to a large number of halo signs in MR images. It was found that the grading of periportal halo signs helps to distinguish stage II versus III, and stage III versus IV, but not stages I and II. Non-homogeneous liver signal intensity reflects the inflammation and cholestasis throughout all stages, and numerous and diffused halo signs in the foundation of non-homogeneous liver signal intensity in stage III and IV made the liver signal intensity more uneven. Thus, our study suggests that the grade of the two signs could be an indicator for evaluating the histological stage and progression of PBC.

Some recent studies have shown that enlarged lymph nodes are a frequent finding in PBC patients, and some research suggest that the size of the lymph nodes is positively related to the progression and chemical indexes reflecting hepatocyte damage[12,16,23,24]. Lymphadenopathy was noted in 88% of patients in the research conducted by Blachar et al. [16], which is similar to our result of 86.7%. Enlarged lymph nodes are T1-weighted low and T2-weighted high signal intensity nodular lesions, and they distribute mainly in the portacaval space, porta hepatis, peripancreatic space, retroperitoneal space, aortocaval space and gastrohepatic ligament. In our study, mildly enlarged nodes were present in 85.8% of patients, and distributed predominantly in the portacaval space and near the porta hepatis, which is similar to the results of Zhang et al. [23], who used a multi-slice CT. MRI provides high-contrast resolution of soft tissues, and it has more advantages to show lymphadenopathy in a T2-weighted image with fat saturation than CT. Lymphadenopathy is an expression of benign hyperplasia[16] and some studies have consistently shown a high percentage of lymphadenopathy with none malignant tumor in our research, suggesting that enlarged lymph nodes are associated with inflammatory reaction, rather than with the malignant tumor metastasis. The inflammatory reaction throughout the entire disease may explain our result of no significant difference among the four histological stages for the occurrence and size of enlarged lymph nodes.

In this study, the rate of lymphadenopathy in PBC was as high as 86.7%, and there was a large difference between PBC and other diffuse liver diseases. One study [23]reported that lymphadenopathy was significantly more common in PBC patients than in hepatitis B-induced cirrhosis patients. Thus, it is necessary to exclude PBC firstly if lymphadenopathy was found in diffuse liver disease without lymphoma or other malignancy.

### The limitations of the study

Liver signal intensity classification and periportal halo sign grading were subjective, and there is lack of quantitative indicators. It has also been widely recognized that MR functional imaging (e.g. DWI, MRS, and MRE) has an important role in the evaluation of the degree of liver fibrosis. However, there is lack of data to evaluate the value of MR functional technology in the diagnosis of PBC, for this research is a retrospective study. Kovač et al. [13] reported that the mean ADCs in PBC patients were significantly different at stage I versus III and IV, and stage II versus IV, and they suggested that DWI could be used for assessment of liver fibrosis distribution and for disease staging. However, the majority of studies usually include patients with liver fibrosis that is caused by common reasons[25–27]. More research needs to be conducted with PBC patients.

Another potential extension of this study is to use a more rigorous and objective method for quantitative assessment of the MR images, which is currently done via grading by experienced readers. There are several established and well accepted methods such as the image texture analysis[28], grey level co-occurrence matrix (GLCM) measurement [29–30], etc. Although these methods may improve the precision of the quantitative assessments, the computational metrics used in these methods may not correspond perfectly to the human visual perception or the routine reading of radiologists. Their application in practice may also not be easily made by radiologists. Nevertheless, inclusion of one of these methods in our future extension of this study may improve the statistical power and aids in the statistical verification.

## Conclusion

MRI reliably detects general signs of PBC, such as hepatomegaly, splenomegaly, widened portal vein diameter, portosystemic collaterals and ascites. Periportal halo sign and lymphadenopathy were specific PBC signs that were identified using MRI. The liver signal intensity classification and periportal halo sign grading were related to the degree of liver fibrosis. In conclusion, MRI is a valuable method in the diagnosis of PBC, and the degree of periportal halo sign and liver signal intensity help to evaluate the degree of liver fibrosis.
